# Fine-tuning Strategies for Classifying Community-Engaged Research Studies Using Transformer-Based Models: Algorithm Development and Improvement Study

**DOI:** 10.2196/41137

**Published:** 2023-02-07

**Authors:** Brian J Ferrell

**Affiliations:** 1 Center for Community Engagement and Impact Virginia Commonwealth University Richmond, VA United States

**Keywords:** fine-tuning, BERT, transformer-based models, text classification, community-engagement, prototype, IRB research, community-engaged research, participatory research, deep learning

## Abstract

**Background:**

Community-engaged research (CEnR) involves institutions of higher education collaborating with organizations in their communities to exchange resources and knowledge to benefit a community’s well-being. While community engagement is a critical aspect of a university's mission, tracking and reporting CEnR metrics can be challenging, particularly in terms of external community relations and federally funded research programs. In this study, we aimed to develop a method for classifying CEnR studies that have been submitted to our university's institutional review board (IRB) to capture the level of community involvement in research studies. Tracking studies in which communities are “highly engaged” enables institutions to obtain a more comprehensive understanding of the prevalence of CEnR.

**Objective:**

We aimed to develop an updated experiment to classify CEnR and capture the distinct levels of involvement that a community partner has in the direction of a research study. To achieve this goal, we used a deep learning–based approach and evaluated the effectiveness of fine-tuning strategies on transformer-based models.

**Methods:**

In this study, we used fine-tuning techniques such as discriminative learning rates and freezing layers to train and test 135 slightly modified classification models based on 3 transformer-based architectures: BERT (Bidirectional Encoder Representations from Transformers), Bio+ClinicalBERT, and XLM-RoBERTa. For the discriminative learning rate technique, we applied different learning rates to different layers of the model, with the aim of providing higher learning rates to layers that are more specialized to the task at hand. For the freezing layers technique, we compared models with different levels of layer freezing, starting with all layers frozen and gradually unfreezing different layer groups. We evaluated the performance of the trained models using a holdout data set to assess their generalizability.

**Results:**

Of the models evaluated, Bio+ClinicalBERT performed particularly well, achieving an accuracy of 73.08% and an *F*_1_-score of 62.94% on the holdout data set. All the models trained in this study outperformed our previous models by 10%-23% in terms of both *F*_1_-score and accuracy.

**Conclusions:**

Our findings suggest that transfer learning is a viable method for tracking CEnR studies and provide evidence that the use of fine-tuning strategies significantly improves transformer-based models. Our study also presents a tool for categorizing the type and volume of community engagement in research, which may be useful in addressing the challenges associated with reporting CEnR metrics.

## Introduction

Community-engaged research (CEnR) is a research approach in which scholars from typical research institutions, such as universities, partner with organizations or members within a community where they share an interest in progressing that community’s well-being. These partnerships bring attention to empirical work in areas that address a range of social, economic, political, and environmental factors that affect health [1]. The problem universities face surrounding CEnR is measuring and reporting these studies due to inadequate methodological processes for reviewing them. This problem leads to unreliable metrics for funders and stakeholders, which leads to a lack of appropriate infrastructure for capturing CEnR data. An example of an appropriate infrastructure would be a system that properly classifies a research study as CEnR or not. Virginia Commonwealth University noticed this problem back in 2013 and began tracking CEnR studies using 3 custom fields in the university’s web-based human subjects protocol submission form (institutional review board [IRB]), as part of an award from the National Center for Advancing Translational Sciences. However, issues arose with these custom fields concerning the quality with which the system documented CEnR studies, such as inconsistent interpretations of the fields by principal investigators submitting protocols.

This led to the exploratory technical study described in another paper [2], which began this process of developing a model for classifying CEnR as a spectrum rather than a binary classification. We sought to automate the classification of a newly created spectrum of CEnR studies using deep learning, which was trained on a small sample of data. After numerous comparisons, we discovered the use of transfer learning to be superior compared to traditional deep learning models. Additionally, we applied the best-performing algorithms to a 5-year data set of unlabeled research protocols (n>6000) to see how well they delineated between the levels of CEnR. The work presented in this paper takes a closer look at the previously trained models and improves them using different fine-tuning methods. In the recent experiment, we found that models generalized better when changing from 6 classes to 3 classes; therefore, the models used in these experiments are trained on 3 different classes.

Transfer learning is a powerful technique for improving the performance of deep learning models. It involves using unsupervised algorithms that have been pretrained on large amounts of unlabeled data to jump-start the learning process for a secondary task. Transfer learning has been shown to be particularly useful for training transformer-based models, which have become popular in recent years due to their ability to process large amounts of data and achieve strong performance on a wide range of tasks [3,4]. However, there is still much to be learned about how to effectively train transformer-based models. Some researchers have found that these models can overfit small data sets and experience catastrophic forgetting, which means that they tend to forget information learned during previous training tasks [5,6]. Several studies have demonstrated that periodically adjusting the learning rate during training can help improve model convergence, as it allows the model to adapt to changes in the data distribution [7,8]. Furthermore, it has been shown that not all layers in a transformer-based model need to be fine-tuned for a given task, and different layers may capture varying levels of syntactic and semantic information [9,10]. Lee et al [11] found that only a quarter of the final layers in their tasks required fine-tuning. These findings have led to the development and implementation of various fine-tuning techniques, such as layer-wise discriminative fine-tuning, gradual unfreezing of layers, layer freezing, and cyclical learning rates, which have been shown to improve model performance [12-17]. Overall, there is a growing body of evidence that fine-tuning strategies can significantly improve the performance of transformer-based models. Further research is needed to fully understand the benefits and limitations of these techniques and to develop more effective approaches for training these models.

The use of transfer learning from our previous experiments resulted in overfitting due to the data set being small combined with the number of parameters from the pretrained models being large. As stated, fine-tuning too aggressively can cause catastrophic forgetting, and fine-tuning too cautiously will slowly lead to overfitting [5]. Therefore, we will observe and make use of some of the previously stated techniques for our problem. We hypothesized that using different fine-tuning strategies for our problem will significantly outperform previously built transformer-based models that classify levels of CEnR. The rest of the paper proceeds as follows: the *Methods* section describes approaches; data collection; data curation; data classification; models; hardware; packages; hyperparameters; train-, test-, or holdout-distributions; model performance tracking; and frameworks for each model trained with its corresponding layer-parameter group. The layer parameter groups provide details on what learning rates were used for each group. The next section is the *Results* section, which provides results across all 3 transformer-based models, showing average *F*_1_-scores, training losses, and so on, followed by the *Discussion* and *Conclusions* sections, which provide principal findings, limitations, and comparisons from previous experiments.

## Methods

### Data

The data were collected from our university’s IRB database. After cleaning and deduplicating the data, we were left with 6000 research studies, of which 360 were pulled, reviewed, and manually labeled as one of the original 6 classes from our previous study. Our training data set is derived from data augmentation techniques on the 360 research studies, creating 2000 contextually similar training samples. Our testing data are then the original, unaugmented data. Because of the augmentations, it is easier for transformer models to do well on the testing set; therefore, we have a holdout data set of 50 research studies. The holdout data sets’ performances are what we report on in the paper. Textbox 1 shows the original 6 classes that represent the levels of CEnR [18]. However, our most recent study allowed us to see that combining the classes to fit a broader spectrum was best. For these experiments, containing just the 3 classes, we collapsed the 1s and 2s (=1), the 3s, 4s, and 5s (=2), and kept the class 0 as is.

**Community-engaged research (CEnR) levels that were used to manually classify the training data.**

0=No CEnR

- Research without a partnership or community engagement

1=Non-CEnR partnership

- There is reference to a partnership, but the relationship is uncategorizable (eg, not adequately described) or not a traditional community-engaged partnership (eg, contractual relationships).

2=Instrumental partnership

- The community partner primarily facilitates access to the “inputs” needed to conduct the study (eg, posting recruitment flyers, providing participant contact information, extracting data, and providing study sites for observation).

3=Academic-led partnership

- There is minimal yet important interaction between the research team and the community partner, which is often essential to project success (eg, academic partners take the lead on study design and research activities, with community partner involvement at particular points, such as troubleshooting recruitment or facilitating community meetings).

4=Cooperative partnership

- Shared investment and mutual consideration between the research team and the community partner, without shared decision-making (eg, community advisory boards that provided input on study design and methodology, reviewed data collection instruments, interpreted findings, informed dissemination plans).

5=Reciprocal partnership

- Community partners and research teams share decision-making power and governance (eg, community-based participatory research, team science, and steering committees with decision-making power).

### Models

#### Overview

These proposed approaches are being compared to a previously done experiment where the number of epochs, sequence length, batch size, etc, were already defined and worked for this problem. The only differences between these experiments and the previous ones are the amount of training, testing, and holdout data, the number of training epochs, and the learning rates for different layers. For comparison, we fine-tune 3 transformer-based models, Bidirectional Encoder Representations from Transformers (BERT), Bio+ClinicalBERT, and XLM-RoBERTa.

#### BERT

BERT was introduced by Devlin et al [19]. This was pretrained on BookCorpus (800 million words) and Wikipedia (2500 million words), and the model’s architecture ensures its advantage in Natural Language Processing tasks because it learns the contextual meanings of words and how each word is being used in a sequence due to its 12 attention heads and 110 million parameters. Additionally, BERT can achieve state-of-the-art results on various tasks for large and small data sets, and it does not need to be trained for more than 2-4 epochs. BERT’s baseline version was used for this.

#### Bio+ClinicalBERT

BERT is pretrained on enormous data sets such as BookCorpus and Wikipedia, and in general, this really can model language well. However, Alsentzer et al [20] studied ways to improve this by using BERT models in a more specific way, such as pretraining with clinical text and discharge summaries. The authors used data from the MIMIC-III (Medical Information Mart for Intensive Care) data base in 2 ways: Clinical BERT (which contains all note types) and Discharge Summary BERT (which contains only discharge summaries) so that tasks with clinical data could be used with a more specific classification language model. They then trained 2 BERT models on the clinical text, where one is initialized from the BERT-base model and the other is initialized from BioBERT [21] (this is the model we chose).

#### XLM-RoBERTa

XLM-RoBERTa was not created for our kind of task; however, it still performed very well in the previous experiment. It was introduced by Conneau et al [22] in 2019 and updated in 2020. This model closely resembles the RoBERTa architecture [23], except that it is a cross-lingual model pretrained on 100 different languages. This type of model is made for cross-lingual transfer learning tasks and was trained on more than 2 terabytes of the CrommonCrawl corpora. It differs from BERT in terms of its tokenization and masking pattern, thus making it an interesting model to compare BERT with.

### Training Details

#### Overview

We used the SimpleTransformers library created by Thillina Rajapakse, which can train and evaluate transformer models (derived from the HuggingFace website) with very few lines of code. Since the input text lengths in our sample data set were longer than the limits for BERT and other transformer models, we used a sliding-window technique, which comes as a tool inside the SimpleTransformer’s library. Therefore, sequences from the data (input sentences) that exceed the “maximum sequence length” will be split into subsets, each equaling the maximum sequence length value. Using this technique, each subset of the sliding window has overlapping values, also referred to as the stride (stride=0.8), resulting in about a 20% overlap between the windows. This process lengthens training time but is preferable to truncating data during training. We trained the models on a single Graphics Processing Unit device (NVIDIA GeForce RTX 2070 with 8GB GDDR6 memory). For inference, we use an Intel Core i7-10750H CPU at 2.60 GHz and 32 GB RAM. Additionally, every model had weights corresponding to a class so that it was equally balanced during the training, ensuring no class was heavily favored. As for evaluation metrics and strategies, we compare both accuracy and the *F*_1_-score, but the *F*_1_-score gives a more balanced view of the performance. After every model is trained, we make predictions on the holdout data set and record the accuracy, *F*_1_-score, class accuracies, and the output of final predictions to compare and run evaluations on the best-performing models. The training data comprises 614 samples of the zero’s class, 645 samples of the one’s class, and 769 samples of the two’s class. The testing set comprises 82 samples of the zero’s class, 51 samples of the one’s class, and 146 samples of the two’s class. The holdout data set comprises 17 samples of the zero’s class, 27 samples of the one’s class, and 36 samples of the two’s class.

#### Discriminative Fine-Tuning Procedure

The models in this experiment use the AdamW optimizer [24]. Therefore, for the different learning rates, we split the layers inside every model into 3 groups. Doing this will be computationally easier; that way, we are not performing hyperparameter tuning for every single layer in a transformer model. As for the layer freezing, the learning rate (α) will just be zero. All the models trained have 12 layers; therefore, we created 3 groups consisting of 4 layers in each group. Table 1 shows that the higher layers had larger learning rate values, while the lower and middle layers had smaller ones. This is in correspondence to what the literature says about layers going from general to specific.

**Table 1:** Parameter groupings.

Layer group parameter	Learning rate value list
All Frozen	Learning rates are 0 for every layer in every group
Up until 8th Layer Frozen	*{*Group 1 and Group 2: 0*}*; *{*Group 3: 3e-5 or 4e-5*}*
Up until 4th Layer Frozen	*{*Group 1: 0*}*; *{*Group 2: 1e-5 or 2e-5*}*; *{*Group 3: 3e-5 or 4e-5*}*
None are Frozen	*{*Group 1: 1e-5 or 2e-5*}*; *{*Group 2: 1e-5 or 2e-5*}*; *{*Group 3: 3e-5 or 4e-5*}*

#### Layer Freezing

We did a gradual unfreezing of the layer groups, but not in the way that might be found in the current literature, where the gradual unfreezing part occurs during the training. By this, we mean we compared models where every group’s learning rate is zero (frozen), then “unfreeze” by training models where 2-layer groups are frozen and continue going until none of the layer groups are frozen, adding up to 45 models per transformer. The model names, epochs, groups and so on, are shown on the Weights and Biases (WandB) websites [25-27].

### WandB

Our models during training are connected to a website called WandB [28], which keeps track of the model and its training parameters and performances such as training time, Graphics Processing Unit usage, custom layer parameters, epochs, loss, and so on. This is an organized way to keep track of the 100+ models trained. We have a separate working environment for each of the 3 models, and within each environment, it has a name related to the specific parameter groupings and is colored by the number of training epochs: 3 (blue); 4 (orange); and 5 (green). Links to this data are provided in the *References* section [25-27]. We define parameter groups, which can also be found in the Table section of each of the 3 WandB websites.

### Ethical Considerations

This study involves a secondary analysis of human subject protocols and therefore did not need an IRB review. The research and ethics presented in this study were approved by the IRB of Virginia Commonwealth University; they were the ones that provided these protocols for us to do our analysis. These studies are anonymous and private to the public; we only worked with the IRB applications, not the research itself.

## Results

**Figure 1:**
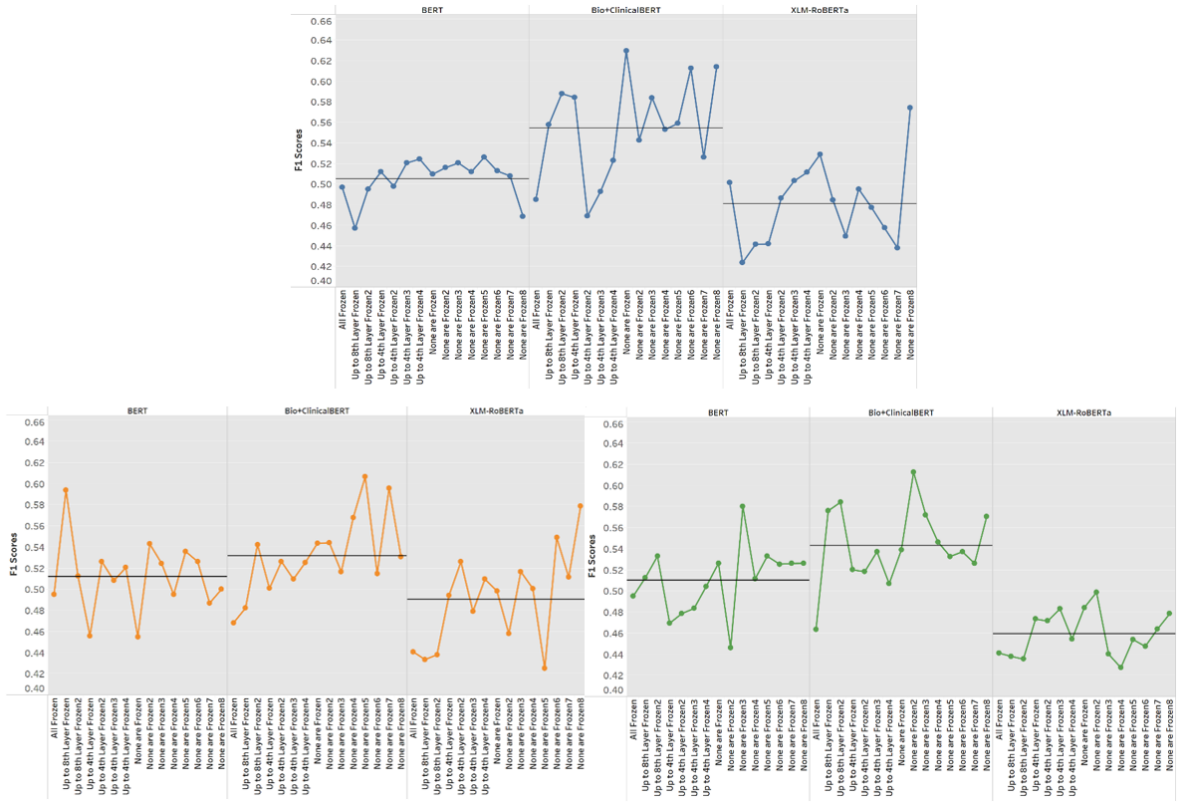
*F*_1_-scores by parameter group (colored by epochs): 3 epochs (blue), 4 epochs (orange), and 5 epochs (green). BERT: Bidirectional Encoder Representations from Transformers.

We performed 3 sets of 45 experiments. In this section, we will discuss the comparisons of all 3 models by their corresponding parameter groupings and epochs. Each table can be seen in Multimedia Appendix 1. The appendix includes the accuracies, *F*_1_-scores, and the accuracies for the individual classes. Figure 1 shows that the transformer model that performed the best overall was Bio+ClinicalBERT, which received the highest accuracy and *F*_1_-score at 73.08% and 62.94% (Table I in Multimedia Appendix 1). BERT and XLM-RoBERTa were able to perform best when training for 4 epochs as opposed to 3 and 5, but Bio+ClinicalBERT achieved a much higher average *F*_1_-score when trained for 3 epochs (as seen in Figure 1). Most of the models do well in the two’s class, averaging about 78%-80% accuracy (most likely due to oversampling in the two’s class); however, they struggle with the ones and zeros. For the one’s class, all the models average around 20%-45% accuracy, and for the zero’s class, they average around 40%-50% accuracy. BERT and Bio+ClinicalBERT outperform XLM-RoBERTa in the one’s class by 10%-20%, but all the transformer models did around the same in the 0’s class. The best parameter group on average was “None are Frozen8,” where all the layer groups had different learning rates (Group 3: 3e-5; Group 2: 2e-5; Group 1: 2e-5), which achieved an average *F*_1_-score of about 54%. The worst parameter group was when all the layers were frozen (no learning rate), achieving an average *F*_1_-score of 46.5%. BERT’s best parameter group was “None are Frozen3,” where the top layers had a much larger learning rate. However, BERT was unpredictable when sifting through the parameter groups. XLM-RoBERTa and Bio+ClinicalBERT start to perform better as more layers become unfrozen, and the upper layers have a learning rate of 3e-5. Their best parameters groups were both “None are Frozen8” (54.3% and 57%).

## Discussion

### Principal Findings

#### Training Losses

WandB also has graphs of each model’s training loss to show how the models performed when grouped by their parameter groups, and WandB has graphs showing the spread across the 100+ models trained, showing their training losses. The only thing worth noting that is distinguishable is that you can see the training losses that stand out the most and have the highest training error. These losses come from the Parameter Group, where all the layers are frozen, with a learning rate of 0. These errors are even more extreme for XLM-RoBERTa [27]. XLM-RoBERTa had a much larger variance in its training loss across models trained for 3, 4, and 5 epochs. The range of loss values is extreme, almost covering the entire graph. Bio+ClinicalBERT [26] was very choppy, with a lot of random spikes, whereas BERT’s [25] training loss was much more smoothed out. The point of this was to show the distribution of training losses across the >100 models that were trained, and we conclude that BERT and Bio+ClinicalBERT did not have as many issues minimizing their error the majority of the time in comparison to XLM-RoBERTa.

#### Comparisons to Previous Experiments

The best Bio+ClinicalBERT model (3 epochs; None Are Frozen) outperformed the best model from the previous experiment with 23% accuracy. In fact, all the models in this paper were higher than the models trained in the previous experiment by 10%-23% in terms of *F*_1_-score and accuracy. Unfortunately, XLM-RoBERTa did not perform as well as we had hypothesized, considering how well it did in the previous work. The prior experiment had 30 samples in the holdout data set, and the models trained were achieving at best 40%-53% accuracy and the same for their *F*_1_-scores; however, with these discriminative fine-tuned models, we were able to achieve about 65% accuracy on average out of that original 30 (+16%) and as high as 63% *F*_1_-score (Table IV in Multimedia Appendix 1). We conclude that discriminative fine-tuning has proved to be better for this text classification task as opposed to training models where every layer has the same learning rate.

### Limitations

The limitations for our experiments still remain somewhat the same as in our previous paper in the sense that our computing power (although significantly increased from the last paper) still has certain computing restrictions. In addition, there was an option for researchers to attach research protocol information in the form of a PDF instead of the database fields, which we did not include in this experiment. We also lack data for the one’s class, so it makes it very difficult for the models to classify those as correct.

### Conclusions

In this paper, we have explored and proposed layer-wise discriminative fine-tuning strategies to improve a previous experiment where we classified newly created levels of CEnR. The contributions of our paper are as follows: we showed a comparative analysis of fine-tuning methods across 3 transformer-based models such as BERT, Bio+ClinicalBERT, and XLM-RoBERTa and how they improve predictive performance, and we compared the specific components of the different strategies mentioned, such as different learning rates for different layers and layer freezing. By conducting a lot of experiments, we have demonstrated that Bio+ClinicalBERT achieved the best *F*_1_-score and accuracy, and most importantly, that transformer models perform better when their learning rates vary for different layers. The reason why Bio+ClinicalBERT outperformed the other transformer models could be because the data contains a lot of clinical studies, and this type of transformer was made to be able to capture the contextuality of clinical data. These performances have significantly improved from prior experimentations, which give us a real opportunity to implement them in a practical system. As for future work, we can continue to explore different strategies such as gradual unfreezing during the actual training, cyclical learning rates, and attaching classification-head architectures to improve these models’ performances, such as a Bidirectional-Long Short-Term Memory Unit.

## Appendix

Multimedia Appendix 1 Results of fine-tuning.

## Abbreviations

BERT: Bidirectional Encoder Representations from Transformers
CEnR: community-engaged research
IRB: institutional review board
WandB: Weights and Biases

## Data Availability

The data sets used during analysis are not publicly available because I have no authorization to make them available. The code and results, however, are publicly available in the form of a multimedia appendix, the WandB website [25-27], and a GitHub repository [29].
