# Supplementary material for: Fine-tuning Strategies for Classifying Community-Engaged Research Studies Using Transformer-Based Models: Algorithm Development and Improvement Study
Source: JMIR Form Res. 2023 Feb 7;7:e41137. doi: 10.2196/41137 (PMC9944122; doi:10.2196/41137)
Supplement: Multimedia Appendix 1 [file formative_v7i1e41137_app1.pdf]

TABLE I
BERT RESULTS (3 EPOCHS)

Model Params	Accuracy	F1 Score	0's	1's	2's
All Frozen	0.6150	0.4970	**0.4799**	0.2500	0.7619
Up to 8th Layer Frozen	0.5960	0.4570	0.3630	0.2500	0.7500
Up to 8th Layer Frozen2	0.6538	0.4950	0.4210	0.2660	0.7990
Up to 4th Layer Frozen	0.6153	0.5120	0.4340	0.3529	0.7500
Up to 4th Layer Frozen2	0.6538	0.4980	0.4440	0.2500	0.7990
Up to 4th Layer Frozen3	0.6538	0.5206	0.3809	0.3749	0.8059
Up to 4th Layer Frozen4	**0.6731**	0.5244	0.4761	0.2857	**0.8116**
None are Frozen	0.6346	0.5096	0.4000	0.3529	0.7761
None are Frozen2	0.6153	0.5160	0.4347	0.3740	0.7380
None are Frozen3	0.6538	0.5206	0.3809	0.3749	0.8060
None are Frozen4	0.6346	0.5119	0.3478	**0.4000**	0.7878
None are Frozen5	0.6538	**0.5262**	0.4210	0.3749	0.7826
None are Frozen6	0.6346	0.5128	0.4211	0.3529	0.7647
None are Frozen7	0.6346	0.5080	0.3630	0.3749	0.7870
None are Frozen8	0.6153	0.4688	0.3636	0.2666	0.7761

TABLE II
BERT RESULTS (4 EPOCHS)

**Model Params**	**Accuracy**	**F1 Score**	**0’s**	**1’s**	**2’s**
All Frozen	0.6346	0.4949	0.4347	0.2500	0.7999
Up to 8th Layer Frozen	**0.6923**	**0.5941**	0.4762	**0.5000**	0.8060
Up to 8th Layer Frozen2	0.6150	0.5125	0.4347	0.3529	0.7500
Up to 4th Layer Frozen	0.5961	0.4560	0.3636	0.2352	0.7690
Up to 4th Layer Frozen2	0.6538	0.5262	0.4210	0.3750	0.7826
Up to 4th Layer Frozen3	0.6346	0.5082	0.4211	0.3157	0.7878
Up to 4th Layer Frozen4	0.6538	0.5206	0.3810	0.3749	0.8060
None are Frozen	0.6346	0.4550	0.4440	0.1428	0.7777
None are Frozen2	**0.6923**	0.5430	**0.5263**	0.2857	**0.8169**
None are Frozen3	0.6730	0.5244	0.4761	0.2857	0.8115
None are Frozen4	0.6538	0.4950	0.4215	0.2666	0.7999
None are Frozen5	0.6538	0.5358	0.4545	0.3529	0.7999
None are Frozen6	0.6346	0.5263	0.4348	0.3749	0.7692
None are Frozen7	0.5962	0.4870	0.3810	0.3529	0.7273
None are Frozen8	0.6346	0.5001	0.4166	0.3076	0.7761

TABLE III
BERT RESULTS (5 EPOCHS)

Model Params	Accuracy	F1 Score	0's	1's	2's
All Frozen	0.6346	0.4949	0.4347	0.2500	0.8000
Up to 8th Layer Frozen	0.6153	0.5125	0.4347	0.3529	0.7500
Up to 8th Layer Frozen2	0.6346	0.5330	0.5000	0.3529	0.7462
Up to 4th Layer Frozen	0.5769	0.4695	0.3333	0.3333	0.7419
Up to 4th Layer Frozen2	0.6346	0.4787	0.3636	0.2666	0.8059
Up to 4th Layer Frozen3	0.6346	0.4835	0.4210	0.2352	0.7941
Up to 4th Layer Frozen4	0.6923	0.5044	**0.5263**	0.1538	0.8333
None are Frozen	0.6346	0.5263	0.4348	0.3749	0.7695
None are Frozen2	0.5769	0.4461	0.3333	0.2666	0.7384
None are Frozen3	**0.7115**	**0.5801**	0.5000	**0.4000**	**0.8405**
None are Frozen4	0.6538	0.5114	0.4545	0.2857	0.7941
None are Frozen5	0.6730	0.5330	0.4444	0.3333	0.8235
None are Frozen6	0.6346	0.5255	0.4545	0.3529	0.7692
None are Frozen7	0.6731	0.5261	0.5000	0.2660	0.8115
None are Frozen8	0.6346	0.5263	0.4347	0.3750	0.7692

TABLE IV
BIO+CLINICALBERT RESULTS (3 EPOCHS)

Model Params	Accuracy	F1 Score	0’s	1’s	2’s
All Frozen	0.7307	0.4850	**0.5882**	0.0000	**0.8684**
Up to 8th Layer Frozen	0.6538	0.5576	0.4348	0.4444	0.7936
Up to 8th Layer Frozen2	0.6730	0.5880	0.5000	0.4705	0.7936
Up to 4th Layer Frozen	0.6730	0.5841	0.4762	0.5000	0.7761
Up to 4th Layer Frozen2	0.6153	0.4690	0.3810	0.2500	0.7761
Up to 4th Layer Frozen3	0.6153	0.4927	0.3636	0.3333	0.7813
Up to 4th Layer Frozen4	0.6346	0.5229	0.4347	0.3529	0.7813
None are Frozen	**0.7308**	**0.6294**	0.5263	0.5333	0.8286
None are Frozen2	0.6731	0.5428	0.4000	0.4285	0.7999
None are Frozen3	0.6538	0.5840	0.4348	0.5556	0.7619
None are Frozen4	0.6346	0.5530	0.4761	0.4444	0.7385
None are Frozen5	0.6731	0.5591	0.4545	0.4285	0.7941
None are Frozen6	0.7115	0.6126	0.5263	0.5000	0.8116
None are Frozen7	0.6538	0.5262	0.4211	0.3749	0.7826
None are Frozen8	0.7115	0.6140	0.4348	**0.5714**	0.8358

TABLE V
BIO+CLINICALBERT RESULTS (4 EPOCHS)

Model Params	Accuracy	F1 Score	0's	1's	2's
All Frozen	**0.7308**	0.4683	**0.5333**	0.0000	**0.8717**
Up to 8th Layer Frozen	0.5962	0.4823	0.3636	0.3333	0.7500
Up to 8th Layer Frozen2	0.6538	0.5424	0.4761	0.3749	0.7761
Up to 4th Layer Frozen	0.6153	0.5009	0.3333	0.4000	0.7692
Up to 4th Layer Frozen2	0.6346	0.5263	0.4347	0.3749	0.7692
Up to 4th Layer Frozen3	0.6346	0.5096	0.4000	0.3529	0.7761
Up to 4th Layer Frozen4	0.6346	0.5255	0.4545	0.3529	0.7692
None are Frozen	0.6538	0.5435	0.4545	0.4000	0.7761
None are Frozen2	0.6538	0.5441	0.4000	0.4440	0.7878
None are Frozen3	0.6154	0.5166	0.3999	0.4000	0.7500
None are Frozen4	0.7115	0.5679	0.4705	0.4000	0.8333
None are Frozen5	0.6923	**0.6066**	0.4545	**0.5714**	0.7941
None are Frozen6	0.6538	0.5149	0.4545	0.3076	0.7826
None are Frozen7	0.7115	0.5958	0.4705	0.5000	0.8169
None are Frozen8	0.6538	0.5307	0.3810	0.4286	0.7826

TABLE VI
BIO+CLINICALBERT RESULTS (5 EPOCHS)

**Model Params**	**Accuracy**	**F1 Score**	**0's**	**1's**	**2's**
All Frozen	**0.7115**	0.4637	0.0000	0.5263	**0.8649**
Up to 8th Layer Frozen	0.6538	0.5760	0.5217	0.4444	0.7619
Up to 8th Layer Frozen2	0.6538	0.5841	0.4348	**0.5556**	0.7619
Up to 4th Layer Frozen	**0.7115**	0.5200	**0.5556**	0.1667	0.8378
Up to 4th Layer Frozen2	0.6538	0.5184	0.4762	0.3077	0.7714
Up to 4th Layer Frozen3	0.6346	0.5373	0.4211	0.4444	0.7463
Up to 4th Layer Frozen4	0.5962	0.5069	0.3810	0.4211	0.7188
None are Frozen	0.6538	0.5390	0.4762	0.3529	0.7879
None are Frozen2	**0.7115**	**0.6126**	0.5263	0.5000	0.8116
None are Frozen3	0.6923	0.5721	0.4762	0.4286	0.8116
None are Frozen4	0.6538	0.5465	0.4348	0.4286	0.7761
None are Frozen5	0.6731	0.5328	0.4762	0.3333	0.7887
None are Frozen6	0.6731	0.5372	0.4000	0.4000	0.8116
None are Frozen7	0.6538	0.5262	0.4211	0.3750	0.7826
None are Frozen8	0.6538	0.5707	0.4545	0.5000	0.7576

TABLE VII
XLM-RoBERTa results (3 Epochs)

Model Params	Accuracy	F1 Score	0's	1's	2's
All Frozen	0.6538	0.5015	0.2105	**0.4706**	**0.8235**
Up to 8th Layer Frozen	0.5962	0.4237	0.3636	0.1429	0.7647
Up to 8th Layer Frozen2	0.6154	0.4415	0.4211	0.1429	0.7606
Up to 4th Layer Frozen	0.6154	0.4419	0.4211	0.1333	0.7714
Up to 4th Layer Frozen2	0.6538	0.4865	0.5263	0.1333	0.8000
Up to 4th Layer Frozen3	0.6538	0.5031	0.4706	0.2500	0.7887
Up to 4th Layer Frozen4	0.6731	0.5114	0.4211	0.3077	0.8056
None are Frozen	0.6538	0.5288	0.3333	**0.4706**	0.7826
None are Frozen2	0.5962	0.4848	0.4000	0.3158	0.7385
None are Frozen3	0.6154	0.4495	0.3158	0.2500	0.7826
None are Frozen4	0.6538	0.4952	0.4000	0.2857	0.8000
None are Frozen5	0.6154	0.4771	0.3333	0.3333	0.7647
None are Frozen6	0.6538	0.4574	0.4000	0.1667	0.8056
None are Frozen7	0.6154	0.4381	0.4000	0.1429	0.7714
None are Frozen8	**0.6923**	**0.5740**	**0.6667**	0.2667	0.7887

TABLE VIII
XLM-RoBERTa results (4 Epochs)

Model Params	Accuracy	F1 Score	0's	1's	2's
All Frozen	0.5962	0.4409	0.2609	0.2857	0.7761
Up to 8th Layer Frozen	0.6154	0.4335	0.3636	0.1429	0.7941
Up to 8th Layer Frozen2	0.6154	0.4381	0.4000	0.1429	0.7714
Up to 4th Layer Frozen	0.6538	0.4942	0.4211	0.2500	0.8116
Up to 4th Layer Frozen2	0.6731	0.5261	0.5000	0.2667	0.8116
Up to 4th Layer Frozen3	0.6154	0.4791	0.4444	0.2500	0.7429
Up to 4th Layer Frozen4	0.6346	0.5097	0.4000	0.3529	0.7761
None are Frozen	0.6538	0.4985	0.4211	0.2857	0.7887
None are Frozen2	0.5962	0.4579	0.3810	0.2353	0.7576
None are Frozen3	0.6731	0.5167	0.3333	0.4000	**0.8169**
None are Frozen4	0.6346	0.5008	0.4762	0.2500	0.7761
None are Frozen5	0.6346	0.4252	0.3158	0.1429	**0.8169**
None are Frozen6	**0.6923**	0.5489	**0.5556**	0.2857	0.8056
None are Frozen7	0.6731	0.5114	0.4211	0.3077	0.8056
None are Frozen8	0.6731	**0.5788**	0.3810	**0.5556**	0.8000

TABLE IX
XLM-RoBERTa results (5 Epochs)

**Model Params**	**Accuracy**	**F1 Score**	**0's**	**1's**	**2's**
All Frozen	0.5962	0.4412	0.2500	0.2857	0.7879
Up to 8th Layer Frozen	0.6154	0.4381	0.4000	0.1429	0.7714
Up to 8th Layer Frozen2	0.6154	0.4355	0.3810	0.1429	0.7826
Up to 4th Layer Frozen	0.6154	0.4734	0.4000	0.2667	0.7536
Up to 4th Layer Frozen2	0.6154	0.4716	0.4000	0.2500	0.7647
Up to 4th Layer Frozen3	0.6346	0.4831	0.4000	0.2667	0.7826
Up to 4th Layer Frozen4	0.6154	0.4545	0.3529	0.2500	0.7606
None are Frozen	0.5962	0.4842	0.3810	**0.3333**	0.7385
None are Frozen2	0.6538	**0.4988**	0.4000	0.3077	0.7887
None are Frozen3	0.5577	0.4401	0.2857	0.3158	0.7188
None are Frozen4	0.5769	0.4273	0.2857	0.2500	0.7463
None are Frozen5	0.6154	0.4540	0.3158	0.2857	0.7606
None are Frozen6	0.6346	0.4475	0.4000	0.1538	0.7887
None are Frozen7	0.5962	0.4639	0.4211	0.2353	0.7353
None are Frozen8	**0.6731**	0.4784	**0.4706**	0.1538	**0.8108**
